# Letters and Answers

**Published:** 1873-10

**Authors:** 


### Our Correspondence.

### Letters Exposing Humbugs and Swindlers,

**With other Communications of Interest to the People.**

Madison, N. Y., Aug. 1st, 1873.

Thad S. Up de Graff, M. D.

*Sir:*—I inclose 50cts for "Bistoury" for 1873. Will you let me know, in a coming number, your opinion. According to Liebig, if it is the way in which we breathe that causes leanness or fat, why is it, then, that lean people generally breathe slow, if *want* of air is the cause of fat? He says that Turkish women have learned to make fat, by their diet of rice, and *enemata* (what does that word mean? is it the same as enema?) of strong soup!

Have you ever heard of a "Mrs. M. J. Brown, *Metephysical* Physician, and can you give me any information about her and her "discovery?" If so, you will do me a great favor. Mrs. C.

1. The number of times a person in ordinary good health, breathes, has no bearing upo the formation of fat. That can be best regulated by diet.

2. The word referred to has the same meaning enema.

3. Mrs. M. J. Brown, "metaphysical physician," and her (?) "discovery" are both shallow frauds. The madam is a cute, male yankee, who has adopted this name as a trade mark to catch ninnies.

Rochester Minn Mar 13 1873.

Mr i inclose one doll, now I want you to keep your Bistoury thair I dont not a nother one if you send a nother i will send it back for your agent lied to me i would not give a cent a year for we have as good a medical men hear as as thair. Mrs. Simeon Smith.

This letter is offered simply to illustrate the class of people that would fail to be interested in a journal like ours. We hope the school masters are as competent "out thair," as the medical men doubtless are.

Dr. A. J. P.—*Waukeenah, Fla.*, is informed that we are not supplied with circulars of the Globe Microscope. The instrument is a very good one for the price. It will accomplish all that is claimed for it in the advertisement.

DALTON, MO., AUG. 12, 1873.

Will you be so good as to send me—either printed in your valuable journal—or written—Brown-Sequard's prescription for "Epilepsy," and oblige. Yours, very truly,

T. A. M., M. D.

The prescription asked for is given here:—

℞

Potassi iodidi, ʒ j ;

Potassi bromidi, ℥ j ;

Ammonii bromidi, ʒ iiss ;

Pot. bicarbonatis, ℈ ij ;

Infusi columbæ, f. ℥ vj. M.

Sig : A teaspoonful before each meal, and three teaspoonfuls at bedtime, in a little water.

---

MEDICAL AND DENTAL HUMBUGGERY.

WESTVILLE, IND., AUG. 1873.

FRIEND UP DE GRAFF:

The last time I troubled you, was in ventilating some traveling quacks who infested northern Indiana like the locusts of Egypt, but they have all migrated to other climes, and we have "Peace in Zion's borders" once more.

I recollect Dr. Chapman used to relate an anecdote of a quite celebrated quack in his day that always carried a stethescope sticking out of his coat tail pocket, but who, the professor informed us, "was himself as deaf as an adder."

Not quite like the above, but as illustrating the "force of appearances," was the case of a now celebrated physician, in one of our western cities, who, becoming reduced in circumstances from indorsing his friend's paper too profusely, removed to a new city, and rented a five hundred dollar house, and sported a buggy and span of fine horses, riding every day over the entire city, and yet without a patient. In remonstrating with him as to his *extravagance*, he informed me that his parlor was the only furnished room in his house, and that it was necessary to success in business to "starve the belly that he might dress magnificently," and to rent a fine house, that he might seem prosperous, for "everything in this world, thee knows, is judged by appearances." And rightly he judged. He soon went into a lucrative practice, when, had he retired to the uburbs and rented a *cheap* house, he would, under his straightened circumstances, have starved to death.

And just here I am reminded of a new dentist who has come into our neighborhood. He has a fine "kit of tools," a hundred and seventy-five-dollar-low-base-Harris chair, that he spends most of his time in showing his visitors, can be moved north, south, east, and west, up or down, horizontally or at any angle, and will turn round on a pivot, like a swing at a circus. He gives them all a free ride, "gratis for nothing," and the remainder of his time is spent in showing the wonderful uses of his sixty dollar, new Morrison engine, and generally ends in applying a corundum burr, and polishing off the tartar from a left superior incisor—but the fun of the thing occurs next day when the patient calls on his own dentist and enquires why his new *white tooth* is so *sensitive* to the air, and the action of hot and cold drinks. The new engine has removed the enamel as well as the tartar. Said dentist has only been in town two weeks, and boasts he filled one hundred and fifty dollars worth of teeth the first week, and from twenty-five to fifty dollars at filling, per day, since, at from three to twenty-five dollars per cavity, when the truth is, the poor devil has neither filled or extracted a single tooth since he opened office. Yet his fine instruments—his magnificent chair, and his novelty engine will *take* with the people, and in due time bring him business, if from no other cause, for the sake of a free ride on the low base one hundred and seventy-five dollar Harris chair.

And now I must refer to a saloon keeper of our town, who, some time since, rented his billiard tables and bar, and, cleaning out his wife's soap kettle, went into the back yard, and by the help of Chase's receipt book, commenced the business of pharmacy. In due time he had a medicine for dyspepsia, another for rheumatism, one for consumption, another for neuralgia, one for liver complaint, one for kidney affections, and another for fits ; with the addition of some liver pills, cathartics, emetics, &c., &c., and a hypodermic syringe, he started forth as a traveling doctor. I am telling you "an o'er true story," not exaggerating or "setting down aught in malice." Finding railroad towns brought him too frequently in contact with friends who had often drank at his bar, he struck back into the country as far from railroads as possible.—His hair which was turning grey, he had always kept daily blackened, but knowing that age favored his new vocation, he permitted it to assume its normal color, and now you may see his bills headed *Old Doctor* Pilgarlic will visit so and so. He cures fits in two days, headache in fifteen minutes, consumption in three to ten days, &c., &c., and this doctor is a fair specimen, and among the best and most learned of the traveling fraternity.— When will the people learn wisdom ?

It is told of a celebrated London quack who made a fortune and retired, on giving a party to his professional brethren, was asked by one of his guests what was the secret of his success ? The doctor told him to notice particularly the crowd of people who passed the window for a few minutes. In due time he enquired of his guest how many *intelligent* looking persons had passed the window. He replied, one or two in a hundred. "You learned doctors," said he, "get the one or two patients, we quacks the hundred." And this reminds me of a graduate of a Cincinnati Medical school, twenty or thirty years ago, now a retired wealthy man, whose favorite prescriptions were for a cough powder:—R. Pulv. opii. sulp. morph. dovers powder, equal parts. That prescription might be seen in any drug store, and it stopped the cough suddenly; and one other: R. Comp. ext. clocynth ℈i, calomel, and pulv. rhubarb, grs. xv. mix and make into five pills. The druggists used to complain they were pretty large. I never knew what the patients said of them, but concluded they were like the witness' stone, as large as a piece of chalk. But enough for the present. Yours, D. L. T., M. D.

The Doctor's letter is self explanatory, and needs no remarks. The country is full of just such adventurers as he aptly describes. People always have, and we suppose always *will*, patronise them, to the exclusion of reliable practitioners in their own midst.

---

Dr. R. A.—*Millford, Conn.*—The foot cannot be straightened by the apparatus alone. Cut the *tendo achillis.*

---

CHEATHAM Co., TENN. Sept. 4th, 1873.

EDITOR BISTOURY :

*Dear Sir.*—Having by chance seen a BISTOURY this morning, and reading your kind answer to the afflicted, I have determined to ask you to help me, although I must do it *secretly*, for having once given $100 to a humbug, my father frowns at the mention of a "*sure cure for deafness.*" I am 18 years of age ; when about 13, I had mumps very badly. We think cold taken at the time caused my deafness ; when I take cold my hearing is worse, (I am only deaf in one ear—the left) can hear better on a clear frosty morn. I can hear a watch tick when placed on any part of my head, except on the end of my nose, and then I can hear no sound at all. Have a roaring in my head as that of distant waters and bells. Although we live within 1½ mile of the river, I have not heard the sound of a boat for seven years.

Physicians tell me it is a thickening of the membrane of the ear. Have good health. I will wait very patiently for a reply, and can but hope for the best. Please help me, if you can. MISS M. A. M.

We give this letter in full, as a sample of many similar ones which we are constantly receiving. We desire to assure the young lady, and all similarly afflicted that no relief can be found in advertised nostrums, recommended for the cure of deafness. Neither can we or any other physicians, prescribe a course of treatment that can be conducted at home, by means of correspondence.

The patient must place herself under the care of some competent aurist, and remain under his daily observation for perhaps months before a cure, if ever, can be effected. Any aurist of acknowledged ability can decide, after a suitable examination of the hearing apparatus, whether any benefit can be expected from a course of treatment. Consult such a person and abide by his decision, but do not throw your means away upon traveling mountebanks.

---

WELLS NEAR GRAVE YARDS.—Mons. Lefort has made analyses of water taken from wells in the neighborhood of graveyards, and finds the water of wells situated 150 feet from graveyards to have a sweet, fetid taste, and upon evaperation by heat, a thick greyish residue remained, which had a strong empyreumatic oder. Upon adding reagents, carbonic acid was given off, and salts of ammonia were thrown down. He thinks that no bodies of men or animals should be buried nearer than 300 feet to any well from which water is taken for household purposes.

